# A new mathematical approach for qualitative modeling of the insulin-TOR-MAPK network

**DOI:** 10.3389/fphys.2013.00245

**Published:** 2013-09-12

**Authors:** H. Frederik Nijhout, Viviane Callier

**Affiliations:** ^1^Department of Biology, Duke UniversityDurham, NC, USA; ^2^School of Life Sciences, Arizona State UniversityTempe, AZ, USA

**Keywords:** mathematical model, sigmoid, insulin, TOR, MAPK, FOXO, growth

## Abstract

In this paper we develop a novel mathematical model of the insulin-TOR-MAPK signaling network that controls growth. Most data on the properties of the insulin and MAPK signaling networks are static and the responses to experimental interventions, such as knockouts, overexpression, and hormonal input are typically reported as scaled quantities. The modeling paradigm we develop here uses scaled variables and is ideally suited to simulate systems in which much of the available data are scaled. Our mathematical representation of signaling networks provides a way to reconcile theory and experiments, thus leading to a better understanding of the properties and function of these signaling networks. We test the performance of the model against a broad diversity of experimental data. The model correctly reproduces experimental insulin dose-response relationships. We study the interaction between insulin and MAPK signaling in the control of protein synthesis, and the interactions between amino acids, insulin and TOR signaling. We study the effects of variation in FOXO expression on protein synthesis and glucose transport capacity, and show that a FOXO knockout can partially rescue protein synthesis capacity of an insulin receptor (INR) knockout. We conclude that the modeling paradigm we develop provides a simple tool to investigate the qualitative properties of signaling networks.

## Introduction

Signaling through the insulin pathway is a major regulator of growth in a broad diversity of vertebrates and invertebrates ranging from humans and mice, to worms and flies (Oldham and Hafen, 2003; Taniguchi et al., 2006; Sutter et al., 2007; So et al., 2011). It regulates diverse processes such as blood glucose homeostasis, differentiation, growth, and senescence (Tatar et al., 2003). The pathway is also frequently misregulated in diseases such as diabetes and different types of cancers. In some cells, insulin regulates the uptake of glucose (Furtado et al., 2003), in others it is a general regulator of protein synthesis and cell growth (Colombani et al., 2003). Among the great discoveries is the interaction between insulin signaling and the Target of Rapamycin (TOR), which mediates between the insulin signal and the utilization of amino acids in protein synthesis and growth (Brogiolo et al., 2001; Oldham and Hafen, 2003; Avruch et al., 2006; Grewal, 2009; Kim and Guan, 2011). The insulin network also interacts with members of the FOXO family of transcriptional regulators in the control of cell growth and cell proliferation (Jünger et al., 2003; Puig et al., 2003; Southgate et al., 2007; Tang et al., 2011; Snell-Rood and Moczek, 2012). The insulin signal is able to stimulate upstream members of Ras/Raf-mediated MAPK cascades (Seger and Krebs, 1995), which also lead to cellular growth.

Experimental work to understand the properties and function of the insulin signaling network has tended to focus on the roles of one or two components of the network at a time, for example by knocking out or overexpressing one component and examining the phenotypic effect. The overall network is a conceptual framework built up of a large set of individual experiences and understandings. The network is becoming increasingly complex and it is difficult to know how the various components interact with each other. Experimentally, it is impractical, and in most cases impossible, to control for all potential variables, nor is it possible to determine to what degree an experimental result is a consequence of the particular background in which it was performed. Indeed, the functional consequence of a mutation is largely dependent on the background in which it occurs (Dworkin et al., 2009; Chandler et al., 2013). Alternatively, if the experiment was done under highly controlled conditions, *in vitro*, on a subset of the system, it is not possible to say how that subset would actually perform when embedded within the more complex network with all its many inputs, interactions, and feedback loops. With very simple systems it is possible to perform thought experiments that examine the logic of a network diagram by simply stepping through it. But when the system is large, with positive and negative feedback loops and a mixture of activating and inhibitory interactions, thought experiments generally fail. In addition, although our understanding of the components and overall structure of signal transduction networks is growing rapidly, the dynamical properties of these networks remain largely unknown. This is important because the explanation of some cellular phenomenon lies not in the components but in the dynamics of the system that led to the phenomenon (Wolkenhauer et al., 2004).

A grasp of mechanics and combinatorial possibilities of interactions between signaling molecules is insufficient for understanding cellular responses or changes in physiological states. This is because the set of interactions that actually make a functional difference to the cell are a small subset of all the molecular interactions that occur; furthermore, detailed knowledge of local interactions may not be sufficient to understand the global dynamics of the network. Uncovering which components and which dynamics are functionally important is one of the most challenging and important questions for understanding the function and evolution of these signaling networks.

One solution to understanding the properties of a complex network is through mathematical modeling. A mathematical model is nothing more than a quantitative, simplified abstraction of the structure and kinetics of the system. It has two advantages: one is that a mathematical model is completely explicit in what is included and what is not, something an experimental system seldom if ever can be, and second that it allows one to examine whether the network elucidated by experimentation indeed has the properties we assume it does.

There are many approaches to mathematical modeling. Boolean models can describe the logic of a regulatory network, differential equation models can describe the kinetics of a network, and statistical models can describe the patterns of correlation and covariance within a network. Several investigators have developed differential equation-based mathematical models for different portions of the insulin signaling network (Sedaghat et al., 2002). Some of these models reproduce selected experimental data well (Sedaghat et al., 2002), while others do not attempt to do so and are mostly concerned with the general or the formal properties of the model mechanism (Alon, 2006; Vinod and Venkatesh, 2009).

The most common problem encountered in building a mathematical model is the dearth of data on the kinetics of the reactions. Most data are static and record the response to an insulin stimulus as “fold-activation” or “percent of control value” or “percent of maximum response.” Thus response data are typically reported as scaled quantities. The modeling paradigm we develop here uses scaled variables and is ideally suited to simulate systems in which much of the available data are scaled. We develop a simple network modeling paradigm, inspired by Boolean, genetic, and neural network models (Reinitz and Sharp, 1995; Vohradsky, 2001; Jaeger et al., 2004; Faure et al., 2006; Martin et al., 2007), that can be used to study the qualitative behavior of complex networks, and apply it to a study of the insulin signaling network. Abstracting the network in a way that is consistent with the available data opens possibilities to reconcile experiments with theory, and thus improves our understanding of the structure and function of signaling networks. A well-validated mathematical model is also a useful adjunct tool for the experimental biologist because it allows for quick and inexpensive testing of alternative hypotheses and may provide suggestions for experimental design.

## Methods

### Structure of the network

**Figure 1:**
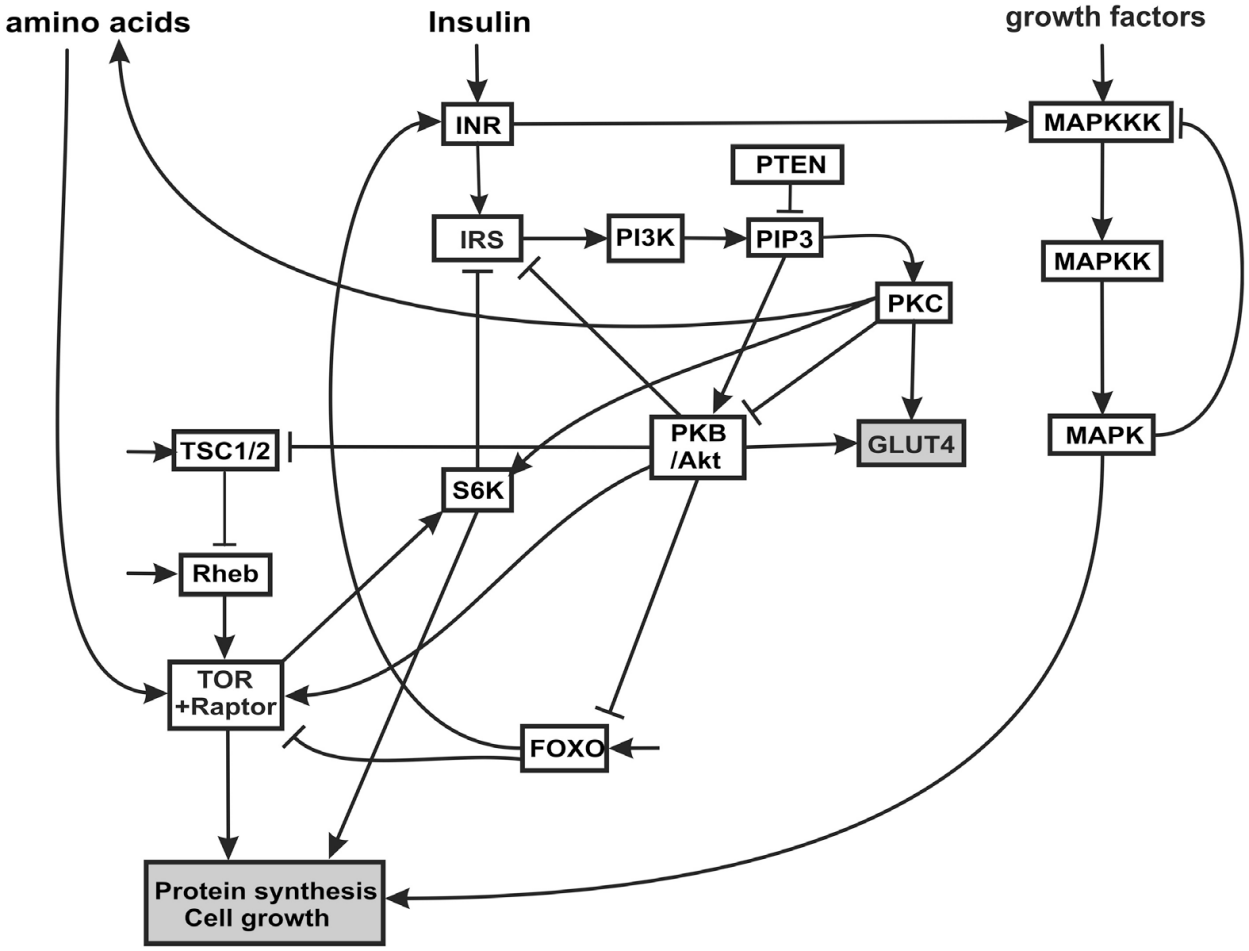
**Insulin and MAPK signaling network**. The acronyms for the components of the network and the rationale for the connectivity are explained in the text.

The insulin signaling network we model is illustrated in Figure 1. Insulin binds the insulin receptor (INR), causing it to become phosphorylated and thus activated (Oldham and Hafen, 2003). INR has multiple phosphorylation sites which become autophosphorylated upon binding of insulin. The activated INR recruits Insulin Receptor Substrate adaptor proteins (IRS) to the cell membrane. The IRS proteins bind to phosophoinositide 3-kinase (PI3K) and to phosphatidylinositol(3,4,5)-triphosphate (PIP3), and recruit both to the membrane. Biochemically, PI3K becomes activated when it interacts with specific phosphotyrosine motifs in the IRS (Taniguchi et al., 2006). Activated PI3K phosphorylates phosphatidylinositol(4,5)-biphosphate (PIP2, also at the cell membrane) into PIP3. The tumor suppressor gene phosphatase tensin homolog deleted on chromosome 10 (PTEN) catalyzes the opposite reaction, dephosphorylating PIP3 to PIP2. PTEN reduces the amount of PIP3, a substrate necessary for the activation of protein kinase B (PKB, also called Akt) (Brazil and Hemmings, 2001). Through this mechanism, PTEN is an antagonist to insulin signaling. PTEN is one of the most commonly lost tumor suppressor genes in human cancer. It has relatively high constitutive phosphatase activity (Leslie and Downes, 2004). PIP3 facilitates the phosphorylation of protein kinase C (PKC) by PDK1, thus activating PKC (Standaert et al., 2001; Taniguchi et al., 2006). PIP3 also causes an increase in autophosphorylation of PKC, independent of PDK1, possibly by inducing conformational changes in PKC (Standaert et al., 2001). PKC phosphorylates p70 ribosomal kinase S6K (Valovka et al., 2003). S6K is involved in the regulation of cell cycle and growth (Montagne et al., 1999; Valovka et al., 2003). S6K phosphorylates the ribosomal protein S6, and controls the translation of a class of mRNAs that encode ribosomal proteins and elongation factors (Jefferies et al., 1997).

PIP3 phosphorylates and activates PKB/Akt. PKB has several targets including FOXO, a constitutively active transcription factor (Southgate et al., 2007) that is involved in the cellular response to nutritional conditions (Gershman et al., 2007). When FOXO is phosphorylated by PKB, it is translocated from the nucleus to the cytoplasm, where it can no longer activate transcription (Essers et al., 2005; Aoyama et al., 2006). Under extreme starvation conditions, FOXO upregulates the expression (but not the activation) of the INR, increasing cell sensitivity to insulin, and allowing a fast response to insulin after feeding (Jünger et al., 2003; Puig and Tijan, 2005).

PKB also activates TOR, a serine/threonine kinase that regulates growth in response to nutritional conditions. TOR is required for response to amino acids (Hara et al., 1998). TOR is activated by PKB and positively regulates cell growth via two principal targets, S6K and 4E-BP (Hay and Sonenberg, 2004; Sarbassov et al., 2005). S6K also phosphorylates the INR, decreasing the interaction of INR with its substrate (IRS) and inhibiting insulin signaling (Harrington et al., 2005). The proximal regulator of TOR is a small GTPase, Ras homology enriched in brain (Rheb), which binds to the TOR catalytic domain and activates TOR (Avruch et al., 2006). Amino acid withdrawal interferes with the interaction of Rheb and TOR-raptor, indicating that the Rheb-TOR interaction is responsible for the sensitivity of the TOR pathway to the presence or absence of amino acids. Rheb is negatively regulated by the Tuberous sclerosis complex proteins, composed of TSC1 (hamartin) and TSC2 (tuberin) (Manning and Cantley, 2003). The disease known as tuberous sclerosis is an autosomal dominant disorder associated with benign tumors that is the result of inherited mutations in the TSC1 or TSC2 genes. PKB phosphorylates TSC2 (Choo et al., 2006) inhibiting the function of the TSC1-TSC2 complex (Choo et al., 2006). Thus PKB signaling releases the inhibition of Rheb and activates TOR signaling. The TSC complex is necessary for the downregulation of TOR in response to hypoxia (Ellisen, 2005).

Many growth factors signal through the MAPK cascade. The MAPK cascade is a highly conserved signaling pathway and is a major regulator of growth and differentiation. MAPK cascades typically have three levels (Huang and Ferrell, 1996). The terminal member of the cascade is a MAPK (e.g., ERK, JNK, p34, p42), which is activated by a MAPK kinase (MAPKK: e.g., MEK, EKK), which in turn is activated by a MAPKK kinase (MAPKKK; e.f Raf, Mos). The MAPKKK can be activated in several ways: most commonly by external signals such as epidermal growth factor (EGFR) via a G-protein-coupled receptor complex, and also by insulin signaling (Oldham and Hafen, 2003). The terminal MAPK translocates to the nucleus where it phosphorylates transcriptional regulators for protein synthesis, growth and differentiation.

### Structure of the model

In modeling this system we omit consideration of multiple phosphorylation steps, equilibrium reactions between kinases and phosphatases, and translocations between cytosolic and nuclear compartments. We consider only the activity level of the various kinases and other components in the network. Unlike a Boolean network, in which each element is either on or off, each element in our scheme can have a continuum of activity between zero (inactive) and one (maximum activity). The activity level of a node in the network is a function of activating and inhibitory inputs. We assume that activation follows a sigmoid trajectory, with little activity at low input levels and saturating at high input levels.

**Figure 2:**
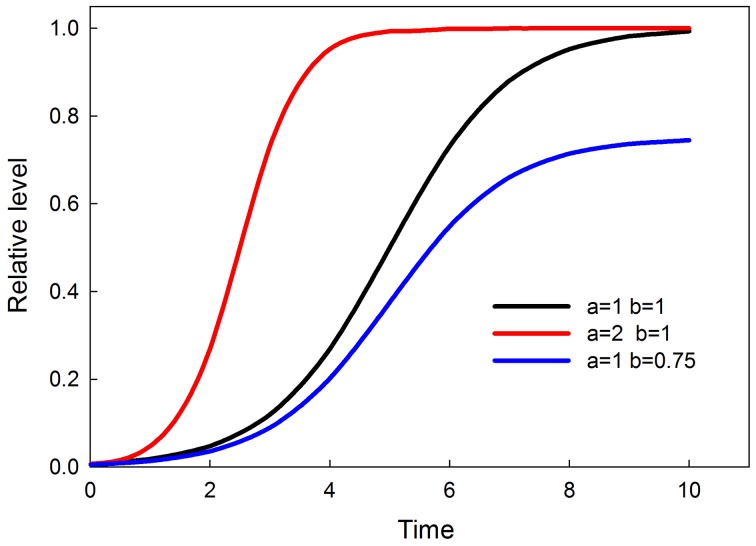
**Time-dependent sigmoids (effect of parameters *a* and *b*)**. Parameter *a* controls when the response attains saturation. Parameter *b* controls the saturation point of the response.

**Figure 3:**
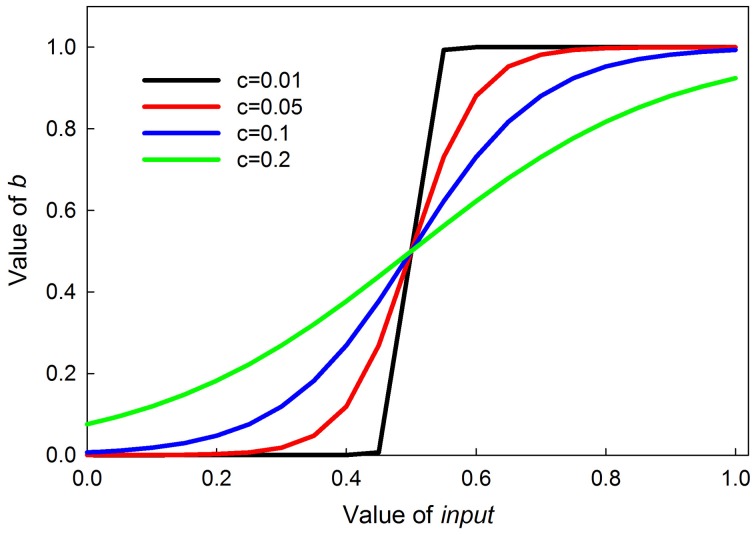
**Graphs of the value of *b* in Equation (2) as a function parameter *c***. Parameter *c* controls the steepness of the sigmoid; the smaller *c* is, the steeper the sigmoid.

The time-dependent activation equation for each node in the network is the logistic

(1) $$\frac{dy}{dt}=ay(1-y/b)$$

where *a* is the rate of increase and *b* is the ceiling. Over time *y* levels off at the value of *b*. Graphs of this time-dependent response, and the effects of parameters *a* and *b*, are shown in Figure 2. The value of *b* is a sigmoidal function of input, so that at low input *b* (and consequently the maximum value of *y*) is small and rises sigmoidally to a value of 1 as input increases. The equation for *b* is a solution to the logistic, as follows

(2) $$b=\frac{1}{1+e^{-\frac{\text{input}-0.5}{c}}}$$

where the 0.5 ensures the inflection point is at an input of 0.5, and *c* sets the steepness of the transition, with smaller values producing a more switch-like transition. The curves are symmetrical around the inflection point. Graphs of this function for different values of *c* are shown in Figure 3.

This pair of functions thus scales the value of *y* between 0 and 1, for a range of inputs that are scaled from 0 to1. The value of *y* then becomes part of the input to the next step in the network. Multiple (activating and inhibiting) inputs are summed as follows: activators are averaged, and inhibitors are averaged and subtracted from the activator mean.

Thus the input function looks like:

(3) input=1n∑i=1nWia∗ctivatori−1m∑i=1mWii∗nhibitori,

where *W*_*i*_ represent the weight of each activator or inhibitor. The weights were chosen in such a way that the model reproduced experimental data (Sedaghat et al., 2002; Danielsson et al., 2005). Values for the weights used in the present model, and the values of all other parameters, are given in Table 1. There is no information available on how multiple inputs are integrated, so we assume a linear weighing scheme. The linear weighing scheme is therefore a hypothesis about how things could work, and as more data become available it might have to be modified. We show that the selection of weights and parameter values in Table 1 is also sufficient to enable the model to reproduce a broad diversity of experimental results.

**Table 1:** **Parameter values and input functions used in the model**.

**Variable**	**Activators**	**Inhibitors**	**Input function**	***c***	***a***
INR	Insulin, FOXO		(insulin.^*^1+ FOXO.^*^0.144)./2	0.15	1
IRS	INR	PKB, S6K	INR.^*^2−(0.1.^*^PKB + 0.2.^*^S6K)./2	0.15	1
PI3K	IRS		IRS.^*^1	0.2	1
PIP3	PI3K		PI3K.^*^1	0.2	1
PKC	PIP3		PIP3.^*^1	0.25	1
PKB	PIP3	PKC	PIP3.^*^1−0.5.^*^PKC	0.15	1
GLUT4	PKC, PKB		(PKC.^*^3 + PKB.^*^4)./2	0.05	1
TSC	Constitutive	PKB	1–0.7.^*^PKB	0.1	0.5
Rheb	Constitutive	TSC	1–0.5.^*^TSC	0.1	1
TOR	Rheb, amino acids, PKB	FOXO	(rheb.^*^3 + 1.^*^aminoAcids + 2.^*^PKB)./3–2^*^FOXO)	0.1	1
S6K	TOR, PKC		(TOR.^*^1.5 + PKC.^*^0.5)./2	0.1	1
Protein synthesis	TOR, S6K, MAPKKK		(S6K.^*^1 + TOR.^*^1 + MAPK.^*^1.2)./3	0.1	1
FOXO	Constitutive	PKB	1–0.9.^*^PKB	0.1	0.5
MAPK	MAPKK		MAPKK	0.1	1
MAPKK	MAPKKK		MAPKKK	0.15	1
MAPKKK	INR, EGF growth factors	MAPK	(egfsignal + PI3K)/2–0.3.^*^MAPK	0.2	1

The model consists of a set of coupled equations of the form of Equation (1), one for each node in the network, with the values of *b* represented by Equation (2) and the inputs by Equation (3). Most nodes are inactive unless activated, with the exception of TOR, Rheb and TSC, which are constitutively active unless inhibited. There are three external inputs: insulin, amino acids, and growth factors that activate the MAPK cascade.

## Results and discussion

### The MAPK cascade and switch-like behavior

**Figure 4:**
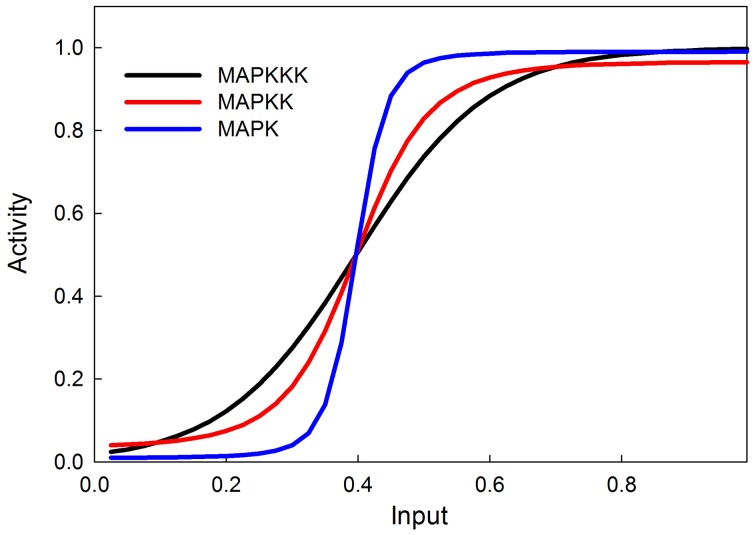
**MAPK cascade simulation shows that the signal becomes more switchlike as it travels down the MAPK cascade, as shown in Huang and Ferrell (1996)**.

**Figure 5:**
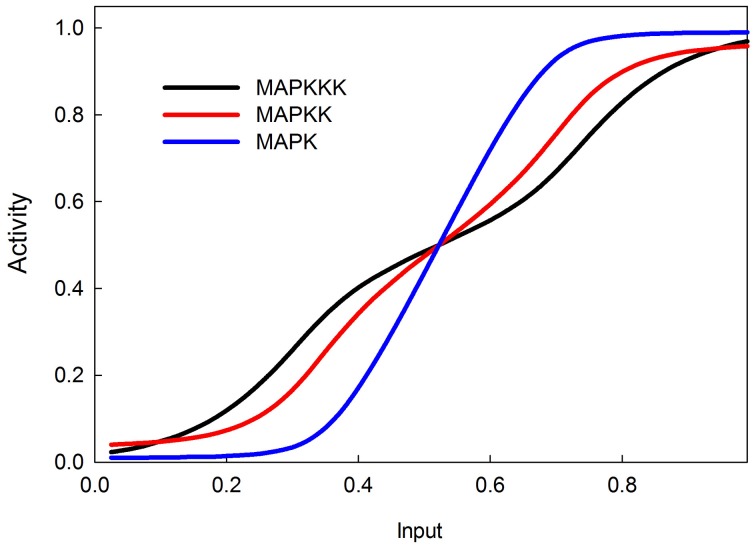
**MAPK cascade with negative feedback from MAPK to MAPKKK**. The negative feedback makes the response less switchlike.

The MAPK phosphorylation cascade is one of the most prevalent signal transduction pathways, typically mediating between a G-protein coupled surface receptor for a growth signal and a transcriptional regulator that affects growth and cell proliferation. The MAPK cascade can also be activated by insulin signaling via the stimulation of the upstream kinase (e.g., Raf) via INR (Oldham and Hafen, 2003). MAPK cascades have either three or four levels with multiple phosphorylation steps at each level (Huang and Ferrell, 1996). This structure sharpens the response to a graded signal and makes the response increasingly more switch-like at successively lower levels of the cascade (Huang and Ferrell, 1996). In our model we do not explicitly model phosphorylation and dephosphorylation steps but instead model the transition between an active and inactive kinase, using our sigmoid formalism. Figure 4 illustrates the behavior of the 3-step MAPK cascade we model to a linear graded input and shows the expected switch-like behavior. The increasing steepness of the response emerges from the fact that each lower step in the cascade is responding to a sigmoidal input in a sigmoidal fashion. MAPK cascades may have a negative feedback regulation by the last to the first member in the cascade (Brondello et al., 1997; Keyse, 2000; Kholodenko, 2000; Asthagiri and Lauffenburger, 2001; Nijhout et al., 2003). Figure 5 shows the dose-response behavior of our model when such a feedback is included, using the same kinetic parameters as in Figure 4. Including the feedback makes the response of the terminal kinase less switch-like.

### Insulin dose-response

In our model insulin concentration can vary from zero (no insulin) to 1 (maximum or saturating insulin). Dose-response curves are typically hyperbolic on linear axes and sigmoidal when plotted on a logarithmic x-axis. In our scheme all axes are linear and the linear x-axis must therefore represent a logarithmic insulin concentration scale. The dose-response of INR phosphorylation to insulin in experimental settings spans about 4-orders of magnitude of insulin concentration (from 10^−11^ to 10^−7^ M) (Stagsted et al., 1993; Kurtzhals et al., 2000; Sedaghat et al., 2002; Danielsson et al., 2005). Thus on our linear scale 0.25 units correspond approximately to one decade on a logarithmic scale.

**Figure 6:**
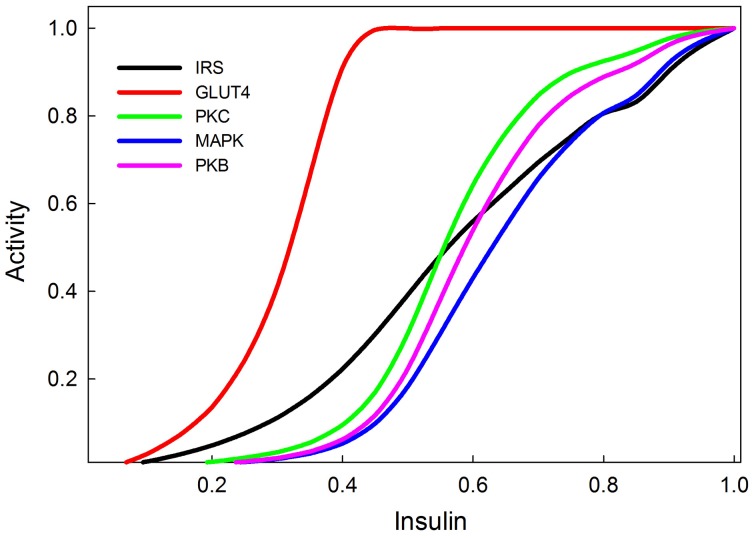
**Insulin dose response curves of various components of the insulin and MAPK signaling pathways replicating (Stagsted et al., 1993; Sedaghat et al., 2002; Danielsson et al., 2005)**. The GLUT4 transporters are the most sensitive to insulin signaling, and achieve their maximum response at relatively low levels of insulin stimulation (0.4).

Dose-response curves for active IRS, active PI3K, PKB, GLUT4, and MAPK, as functions of insulin concentration are shown in Figure 6. The activities are scaled to the maximal response and these relative responses closely resemble the empirical data of Stagsted et al. (1993); Danielsson et al. (2005), except for the response of PKB, which in experimental data appears to saturate at somewhat lower concentrations of insulin than it does in our model.

### Amino acids, protein synthesis, and tor knockout

**Figure 7:**
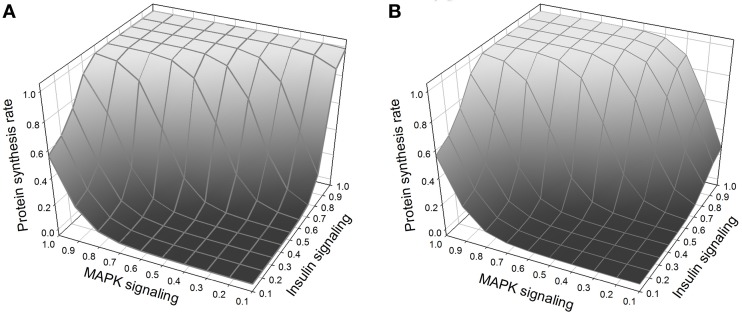
**(A)** Protein synthesis as a function of MAPK and insulin. MAPK and insulin act synergistically, and the maximum protein synthesis occurs when both pathways are activated. Nevertheless, at low levels of MAPK signaling, insulin is still able to stimulate protein synthesis. **(B)** Same figure but with TOR knockout. The effect of the TOR knockout on protein synthesis is only noticeable when there is weak MAPK signaling (right corner of the figure); when TOR is knocked out and MAPK signaling is low, insulin alone cannot stimulate protein synthesis.

Normal growth requires, in addition to insulin signaling, amino acid signaling through TOR (Hay and Sonenberg, 2004; Martin and Hall, 2005; Kim and Guan, 2011). In our model, amino acids activate TOR/Raptor directly. In addition, insulin signaling is known to stimulate the import into the cell of neutral amino acids that are handled by the system-A transporter (Kilberg, 1982; McDowell et al., 1998; Biolo et al., 1999; Jones et al., 2006). The exact mechanism is not fully understood, and here we model it as an effect of PKC. Thus growth is regulated by three interacting pathways, MAPK, insulin, and TOR (Figure 7). MAPK and insulin signaling can stimulate growth autonomously, but TOR requires activation by insulin signaling in order to be sensitive to stimulation by amino acids.

### PTEN, a tumor suppressor gene

**Figure 8:**
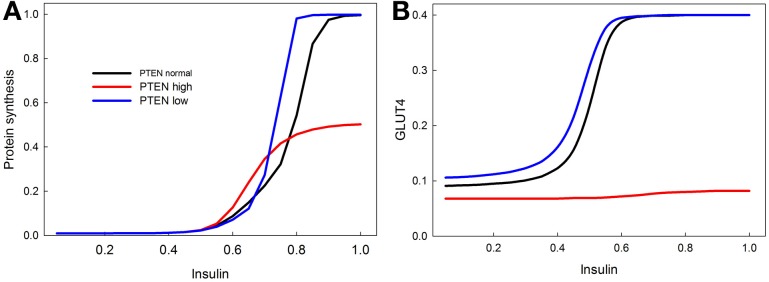
**Effect of PTEN over expression and knock-down on the sensitivity of insulin stimulated protein synthesis (A) and GLUT4 activation (B)**. PTEN overexpression reduces the maximum level of protein synthesis, and completely abrogates the response of GLUT4 to insulin stimulation. PTEN underexpression does not have as dramatic an effect, but does increase the sensitivity of protein synthesis in response to insulin especially at high insulin levels. In contrast, the stimulatory effect of PTEN underexpression on GLUT 4 sensitivity is mainly observed at low insulin levels.

PTEN is a well-known tumor suppressor gene, and many cancers are associated with a reduction in PTEN activity (Leslie and Downes, 2004; Nassif et al., 2004; Song et al., 2012). We show with our model that knocking out PTEN increases protein synthesis at lower insulin levels (Figure 8A), and increases insulin sensitivity of GLUT4 activation (Figure 8B). This is consistent with data showing that PTEN haploinsufficiency increases the probability of developing tumors, and also increases insulin sensitivity (protects against diabetes) (Pal et al., 2013). Overexpression of PTEN, by contrast, greatly reduces both proteins synthesis and GLUT4 activation, consistent with its role as a tumor suppressor (Zhao et al., 2005).

### FOXO: a regulator of phenotypic plasticity?

Insulin signaling regulates growth and size in response to nutrition (Oldham and Hafen, 2003; Grewal, 2009), but few morphological traits scale isometrically: morphological traits commonly scale hyper- or hypo-allometrically with size. These scaling relationships must arise from differential sensitivity of tissues to systemic growth factors and nutritional availability.

Recent evidence indicates that differential trait growth is a consequence of differential sensitivity to insulin, regulated by FOXO (Tang et al., 2011; Snell-Rood and Moczek, 2012; Shingleton and Frankino, 2013). Genitalia of flies (Tang et al., 2011) and of beetles (Snell-Rood and Moczek, 2012) are relatively insensitive to nutrition (or lack thereof). In contrast, wings and horns are more sensitive to nutrition (Tang et al., 2011; Emlen et al., 2012). In beetles, knockdown of FOXO using RNAi causes slight but significant overgrowth of the genitalia (Snell-Rood and Moczek, 2012), indicating that in normal growth conditions, FOXO represses insulin-stimulated growth in that tissue. Loss of FOXO causes genitalia and wings to both scale isometrically with body size (Tang et al., 2011), indicating that FOXO is necessary to maintain trait-specific sensitivity to insulin.

In normal growth conditions, increased expression of FOXO decreases body and organ size (Jünger et al., 2003; Puig et al., 2003). In poor nutritional conditions or in Insulin Receptor (InR) mutants, growth is inhibited, and this reduction in growth is partially rescued by knocking out FOXO (Tang et al., 2011). A similar phenomenon has been observed in *C. elegans*: mutants in Daf-2 (INR homolog) arrest growth at the dauer stage, but null mutations in Daf-16 (FOXO homolog) suppress the effects of mutations in Daf-2, rescuing growth (Ogg et al., 1997). This suggests that in both flies and worms, FOXO knockout rescues InR mutants.

**Figure 9:**
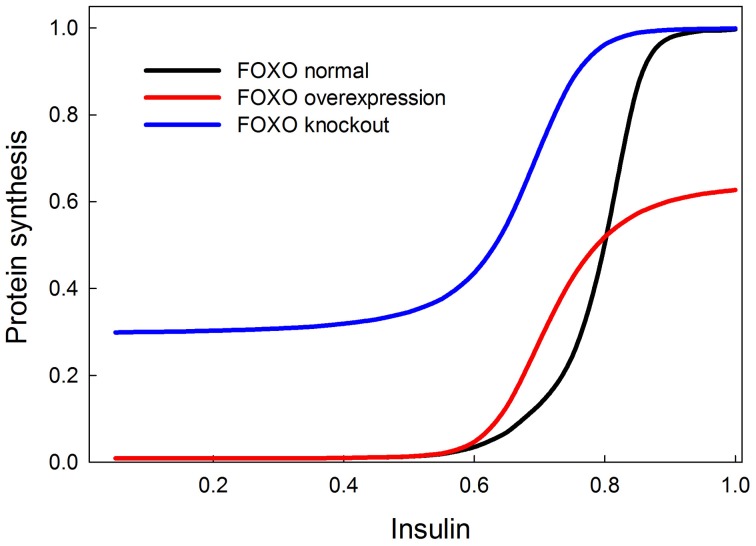
**Effect of overexpression and knockout of FOXO on insulin-stimulated protein synthesis**. FOXO knockout increases protein synthesis especially at low levels of insulin stimulation. On the other hand, FOXO overexpression primarily affects protein synthesis when insulin signaling is high. These two observations can be explained by the fact that FOXO inhibits TOR and this inhibition is inhibited by PKB when insulin is high. At low insulin levels, FOXO should inhibit TOR and protein synthesis, and hence the knockout relieves this inhibition. At high insulin levels, FOXO should be inhibited by PKB and therefore not have an inhibitory effect on protein synthesis, but FOXO overexpression prevents PKB from entirely relieving the constitutive inhibition of protein synthesis by FOXO.

**Figure 10:**
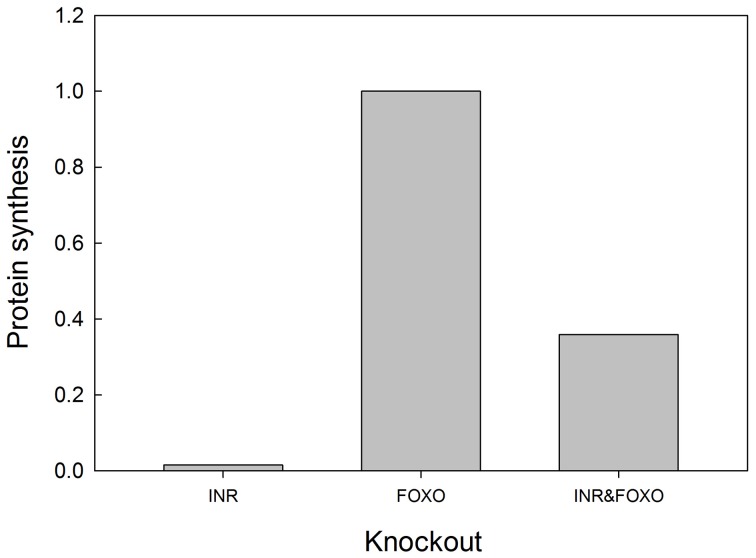
**FOXO knockout rescues the growth defects of INR mutants**. In an INR deficient mutant, protein synthesis is severely depressed and regulated primarily by MAPK (activation of MAPK is set at 0.1 in this experiment). FOXO knockout alone has no effect on protein synthesis, but can partially rescue protein synthesis in an INR knockout. Protein synthesis values are scaled to those of a “wild type.” Input into the MAPK pathway is 0.1.

We tested the model against these predictions. Specifically we tested the following predictions: (1) *FOXO overexpression* should decrease body size/protein synthesis under any nutritional condition. Indeed, Figure 9 shows the result of overexpressing FOXO: protein synthesis is reduced as a function of all insulin inputs, but the effect is particularly strong at high insulin levels (in good nutritional conditions). (2) *FOXO knockout*. Under high insulin signaling conditions/high nutritional conditions, FOXO knockout should slightly stimulate protein synthesis. Under low insulin signaling conditions, FOXO knockout flies should increase protein synthesis relative to wild type flies. Indeed, Figure 9 shows the result of knocking out FOXO. Protein synthesis is increased at low insulin levels, indicating that FOXO knockout compensates for poor nutritional conditions. In high nutritional conditions, protein synthesis is close to wild type, if not slightly enhanced, consistent with the slight increase in size of the beetle genitalia in FOXO knock-down animals (Snell-Rood and Moczek, 2012). (3) *FOXO and InR double knockout*. InR knockout should reduce protein synthesis, and this reduction should be partially rescued by the FOXO knockout. Indeed, Figure 10 shows that protein synthesis is strongly reduced in the InR knockout and that protein synthesis is rescued in the double FOXO-InR knockout. Thus, the model is consistent with previous observations and appears to summarize the developmental mechanism by which FOXO regulates sensitivity to insulin input.

### Hypoxia

Hypoxia decreases body size in a variety of species, although hyperoxia often does not have a symmetrical effect (Harrison et al., 2010; Harrison and Haddad, 2011). The mechanisms by which hypoxia affect growth and size are incompletely understood. Oxygen affects multiple physiological processes and is sensed by a variety of signaling pathways, including the Hypoxia Inducible Factor (HIF) (Gorr et al., 2006) and nitric oxide (NO)/cyclic GMP pathway (Davies, 2000).

HIF and HIF targets interact with components of the insulin signaling pathway; specifically, oxygen affects the inhibition of TSC by Akt (Brugarolas et al., 2004; Deyoung et al., 2008). In normoxic conditions, insulin causes Akt to phosphorylate TSC2. This phosphorylation promotes the binding of TSC2 to its inhibitor 14-3-3 (Deyoung et al., 2008), thereby inhibiting the dimerization and activation of the TSC1/2 complex. In response to hypoxia, REDD1 is induced and sequesters the inhibitor 14-3-3. This releases TSC2 to dimerize with TSC1 and thus promote TSC1/2 function and inhibition of TOR. Thus, in low oxygen conditions, protein synthesis is reduced. Because TSC1/2 is already constitutively active at low insulin signaling, the effects of hypoxia on TSC1/2 will be most apparent at high levels of insulin signaling.

**Figure 11:**
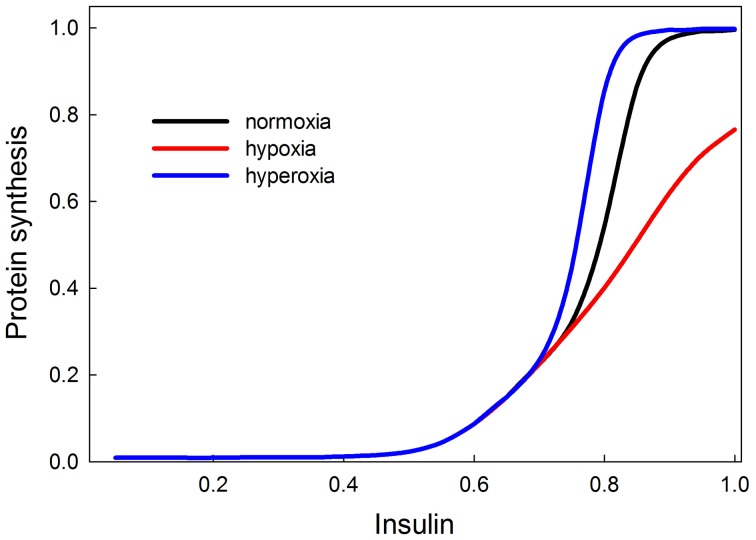
**Effects of oxygen on protein synthesis (growth), mediated by the insulin and TOR signaling pathways are most strongly observed at high insulin signaling levels (>0.7)**. This suggests that in poor nutritional conditions, hypoxia or hyperoxia are unlikely to have strong effects on growth and size; only in good nutritional conditions will oxygen show an effect. Hypoxia decreases the rate of insulin-stimulated protein synthesis. Hyperoxia causes protein synthesis to reach its saturating rate at a slightly lower level of insulin signaling, so it has a slight stimulatory effect for insulin between 0.7 and 0.8. At levels of insulin signaling >0.8, hyperoxia provides no additional stimulation of growth because growth rate has attained its maximum.

Our model shows that low oxygen levels decrease protein synthesis, and the effect is strongest at high levels of insulin (Figure 11). The reason for this effect is that at low levels of insulin, Akt is not stimulated, so Akt is not strongly inhibiting TSC, and the removal of this inhibition has no effect. Hypoxia has the strongest effect on growth when insulin signaling (and Akt) activity is high. In our model, hyperoxia only marginally stimulates protein synthesis beyond its normoxic range. This is consistent with the observation that hyperoxia does not have strong stimulating effects on growth and size in a variety of species (including *Drosophila* and *Manduca*). The fact that the effect of hypoxia is a disinhibition could possibly explain the asymmetrical response of growth to hypoxia and hyperoxia. The double inhibition (Akt inhibits TSC, TSC inhibits TOR) is relieved in hypoxia, but it is unclear whether hyperoxia will significantly enhance the inhibition of TSC by Akt. If this inhibition is already strong in normoxia, then hyperoxia provides no additional benefit. This could explain the asymmetrical growth response to hypoxia/hyperoxia observed in many insect species.

## Conclusions and significance

We have developed a simple and easy-to-implement mathematical model for investigating the logical sufficiency and qualitative behavior of signaling pathways. This model is particularly useful to simulate experimental data that are scaled or normalized, which are the norm in studies of signaling pathways. We use this model to study the behavior of an integrated insulin-TOR-MAPK pathway and compare the results to a broad diversity of experimental data.

Our model provides a simple and easy-to-implement tool for investigating the dynamics of a system that integrates multiple graded inputs and produces a specific output from a set of possible responses. The kinetics of signal transduction pathways are inherently non-linear and our model embraces this nonlinearity by assuming that the strength of a response (e.g., the activation of a kinase), is a sigmoid function of the combined activating and inhibitory inputs, so that at low input there is no response and at high input the response saturates. The model correctly simulates the ultrasensitivity and switch-like behavior of the MAPK cascade. We show that the model also correctly simulates published dose-response curves of INR, PKC, and GLUT4 to insulin input.

We used the model to simulate several experimental observations. TOR has been widely described as a “sensor” for amino acid input and inactivation of TOR by mutation or by rapamycin reduces growth and body size. When we inactivated TOR in our model we found a reduction in insulin-induced protein synthesis (which we use as a proxy for growth), but, as expected, no effect on MAPK-induced protein synthesis. Thus in cases where growth is controlled jointly by insulin and MAPK signaling, the effect of TOR will depend on the relative role of insulin.

Growth depends not only on hormone signaling but also on an adequate supply of amino acids. Amino acids can directly stimulate the TOR branch of the insulin signaling pathway, but insulin signaling also enhances the uptake of certain amino acids. In our model we found a hyperbolic relationship between amino acid availability and S6K activity in the presence of constant insulin signaling, but no unique effect of amino acids on protein synthesis. Protein synthesis required both insulin and amino acids, but increasing amino acid levels did not increase protein synthesis under constant insulin. In accord with experimental findings we found that overexpression of FOXO decreased insulin-stimulated protein synthesis, whereas a FOXO knockout made protein synthesis more sensitive to insulin levels. Likewise, knockout of FOXO can rescue protein synthesis deficiency caused by an INR knockout. Finally, knockout of PTEN and TSC, both well-known tumor suppressors, greatly increased insulin-driven protein synthesis.

The network we simulated here leaves out most of the biochemical details of the insulin and MAPK signaling pathways, and thus can only simulate the qualitative behavior of the network. Even so, the fit to experimental data is excellent (which are typically also qualitative), which suggest that the structure of the network, and the way in which we modeled it, are sufficient to explain the phenomena we studied here.

A natural question that arises when developing a mathematical model is whether all the necessary players and all their interactions have been included. No network is ever complete, and investigators continually find new players and new interactions. But it is not always clear whether each newly discovered item is critical for the normal operation of the network, or whether it is important only under a particular set of conditions, or whether it has only a minor effect. Moreover, investigators often publish small abbreviated models of hypothetical mechanism suggested by their experimental data but seldom investigate whether the proposed mechanism is sufficient to produce the observed behavior. The current modeling paradigm provides a simple tool to do such a test. One of the uses of a mathematical model is to investigate whether such a hypothetical network actually has the expected behavior. A mathematical model can say whether the system that is being modeled is sufficient to produce the observed biological behaviors found under experimental conditions. And when this is not the case, experimentation with the model can often lead the way to discovery of what is missing.

### Conflict of interest statement

The authors declare that the research was conducted in the absence of any commercial or financial relationships that could be construed as a potential conflict of interest.

## References

[B1] Alon U. (2006). An Introduction to Systems Biology: Design Principles of Biological Circuits. New York, NY: Chapman and Hall

[B2] Aoyama H. Daitoku H. Fukamizu A. (2006). Nutrient control of phosphorylation and translocation of Foxo1 in C57BL/6 and db/db mice. Int. J. Mol. Med. 18, 433–439 16865227

[B3] Asthagiri A. R. Lauffenburger D. A. (2001). A computational study of feedback effects on signal dynamics in a mitogen-activated protein kinase (MAPK) pathway model. Biotechnol. Prog. 17, 227–239 10.1021/bp010009k 11312698

[B4] Avruch J. Hara K. Lin Y. Liu M. Long X. Ortiz-Vega S. Yonezawa K. (2006). Insulin and amino-acid regulation of mTOR signaling and kinase activity through the Rheb GTPase. Oncogene 25, 6361–6372 10.1038/sj.onc.1209882 17041622

[B5] Biolo G. Williams B. D. Fleming R. Y. Wolfe R. R. (1999). Insulin action on muscle protein kinetics and amino acid transport during recovery after resistance exercise. Diabetes 48, 949–957 10.2337/diabetes.48.5.949 10331397

[B6] Brazil D. P. Hemmings B. A. (2001). Ten years of protein kinase B signalling: a hard Akt to follow. Trends Biochem. Sci. 26, 657–664 10.1016/S0968-0004(01)01958-2 11701324

[B7] Brogiolo W. Stocker H. Rintelen F. Fernandez R. Hafen E. (2001). An evolutionarily conserved function of the *Drosophila* insulin receptor and insulin-like peptides in growth control. Curr. Biol. 11, 213–221 10.1016/S0960-9822(01)00068-9 11250149

[B8] Brondello J.-M. Brunet A. Pouysségur J. McKenzie F. R. (1997). The dual specificity mitogen-activated protein cascade phosphatase-1 and -2 are induced by the p42/p44MAPK cascade. J. Biol. Chem. 272, 1368–1376 10.1074/jbc.272.2.1368 8995446

[B9] Brugarolas J. Lei K. Hurley R. L. Manning B. D. Hreiling J. H. Hafen E. (2004). Regulation of mTOR function in response to hypoxia by REDD1 and the TSC1/TSC2 tumor suppressor complex. Genes Dev. 18, 2893–2904 10.1101/gad.1256804 15545625 PMC534650

[B10] Chandler C. H. Chari S. Dworkin I. (2013). Does your gene need a background check. How genetic background impacts the analysis of mutations, genes, and evolution. Trends Genet. 29, 358–366 10.1016/j.tig.2013.01.009 23453263 PMC3692003

[B11] Choo A. Y. Roux P. P. Blenis J. (2006). Mind the GAP: wnt steps onto the mTORC1 train. Cell 126, 834–836 10.1016/j.cell.2006.08.025 16959561

[B12] Colombani J. Raisin S. Pantalacci S. Radimerski T. Montagne J. Léopold P. (2003). A nutrient sensor mechanism controls *Drosophila* growth. Cell 114, 739–749 10.1016/S0092-8674(03)00713-X 14505573

[B13] Danielsson A. Öst A. Lystedt E. Kjolhede P. Gustavsson J. Nystrom F. H. (2005). Insulin resistance in human adipocytes occurs downstream of IRS1 after surgical cell isolation but at the level of phosphorylation of IRS1 in type 2 diabetes. FEBS J. 272, 141–151 10.1111/j.1432-1033.2004.04396.x 15634339

[B14] Davies S. A. (2000). Nitric oxide signalling in insects. Insect Biochem. Mol. Biol. 30, 1123–1138 10.1016/S0965-1748(00)00118-1 11044659

[B15] Deyoung M. P. Horak P. Sofer A. Sgroi D. Ellisen L. W. (2008). Hypoxia regulates TSC1/2-mTOR signaling and tumor suppression through REDD1-mediated 14-3-3 shuttling. Genes Dev. 22, 239–251 10.1101/gad.1617608 18198340 PMC2192757

[B16] Dworkin I. Kennerly E. Tack D. Hutchinson J. Brown J. Mahaffey J. (2009). Genomic consequences of background effects on *scalloped* mutant expressivity in the wing of *Drosophila melanogaster*. Genetics 181, 1065–1076 10.1534/genetics.108.096453 19064709 PMC2651043

[B17] Ellisen L. W. (2005). Growth control under stress: mTOR regulation through the REDD1-TSC pathway. Cell Cycle 4, 1500–1502 10.4161/cc.4.11.2139 16258273

[B18] Emlen D. Warren I. A. Johns A. Dworkin I. Lavine L. C. (2012). A mechanism of extreme growth and reliable signaling in sexually selected ornaments and weapons. Science 337, 860–864 10.1126/science.1224286 22837386

[B19] Essers M. A. G. de Vries-Smits L. M. M. Barker N. Polderman P. E. Burgering B. M. T. Korswagen H. C. (2005). Functional interaction between β-catenin and FOXO in oxidative stress signaling. Science 308, 1181–1184 10.1126/science.1109083 15905404

[B20] Faure A. Naldi A. Chaouiya C. Thieffry D. (2006). Dynamical analysis of a generic Boolean model for the control of the mammalian cell cycle. Bioinformatics 22, e124–e131 10.1093/bioinformatics/btl210 16873462

[B21] Furtado L. M. Poon V. Klip A. (2003). GLUT4 activation: thoughts on possible mechanisms. Acta Physiol. Scand. 178, 287–296 10.1046/j.1365-201X.2003.01160.x 12864733

[B22] Gershman B. Puig O. Hang L. Peitzsch R. M. Tatar M. Garofalo R. S. (2007). High-resolution dynamics of the transcriptional response to nutrition in *Drosophila*: a key role for dFOXO. Physiol. Genomics 29, 24–34 10.1152/physiolgenomics.00061.2006 17090700

[B23] Gorr T. A. Gassmann M. Wappner P. (2006). Sensing and responding to hypoxia via HIF in model invertebrates. J. Insect Physiol. 52, 349–364 10.1016/j.jinsphys.2006.01.002 16500673

[B24] Grewal S. S. (2009). Insulin/TOR signaling in growth and homeostasis: a view from the fly world. Int. J. Biochem. Cell Biol. 41, 1006–1010 10.1016/j.biocel.2008.10.010 18992839

[B25] Hara K. Yonezawa K. Weng Q. P. Kozlowski M. T. Belham C. Avruch J. (1998). Amino acid sufficiency and mTOR regulate p70 S6Kinase and eIF-4E through a common effector mechanism. J. Biol. Chem. 273, 14484–14494 10.1074/jbc.273.23.14484 9603962

[B26] Harrington L. S. Findlay G. M. Lamb R. F. (2005). Restraining PI3K: mTOR signalling goes back to the membrane. Trends Biochem. Sci. 30, 35–42 10.1016/j.tibs.2004.11.003 15653324

[B27] Harrison J. F. Haddad G. G. (2011). Effects of oxygen on growth and size: synthesis of molecular, organismal and evolutionary studies with *Drosophila melanogaster*. Annu. Rev. Physiol. 73, 95–113 10.1146/annurev-physiol-012110-142155 20936942

[B28] Harrison J. F. Kaiser A. Vanden Brooks J. M. (2010). Atmospheric oxygen level and the evolution of insect body size. Proc. Biol. Sci. 277, 1937–1946 10.1098/rspb.2010.0001 20219733 PMC2880098

[B29] Hay N. Sonenberg N. (2004). Upstream and downstream of TOR. Genes Dev. 18, 1926–1945 10.1101/gad.1212704 15314020

[B30] Huang C. F. Ferrell J. E. Jr. (1996). Ultrasensitivity in the mitogen-activated protein kinase cascade. Proc. Natl. Acad. Sci. U.S.A. 93, 10078–10083 10.1073/pnas.93.19.10078 8816754 PMC38339

[B31] Jaeger J. Blagov M. Kosman D. Kozlov K. N. Manu Myasnikova E. (2004). Dynamical analysis of regulatory interactions in the gap gene system of *Drosophila melanogaster.* Genetics 167, 1721–1737 10.1534/genetics.104.027334 15342511 PMC1471003

[B32] Jefferies H. B. J. Fumagalli S. Dennis P. B. Reinhard C. Pearson R. B. Thomas G. (1997). Rapamycin suppresses 5'TOP mRNA translation through inhibition of p70 S6K. EMBO J. 16, 3693–3704 10.1093/emboj/16.12.3693 9218810 PMC1169993

[B33] Jones H. N. Ashworth C. J. Page K. R. McArdle H. J. (2006). Expression and adaptive regulation of amino acid transport system A in a placental cell line under amino acid restriction. Reproduction 131, 951–960 10.1530/rep.1.00808 16672359

[B35] Jünger M. A. Rintelen F. Stocker H. Wasserman J. D. Végh M. Radimerski T. (2003). The *Drosophila* Forkhead transcription factor FOXO mediates the reduction in cell number associated with reduced insulin signaling. J. Biol. 2:20 10.1186/1475-4924-2-20 12908874 PMC333403

[B36] Keyse S. M. (2000). Protein phosphatases and the regulation of mitogen-activated protein kinase signalling. Curr. Opin. Cell Biol. 12, 186–192 10.1016/S0955-0674(99)00075-7 10712927

[B37] Kholodenko B. N. (2000). Negative feedback and ultrasensitivity can bring about oscillations in the mitogen-activated protein kinase cascades. Eur. J. Biochem. 267, 1583–1588 10.1046/j.1432-1327.2000.01197.x 10712587

[B38] Kilberg M. (1982). Amino acid transport in isolated rat hepatocytes. J. Membr. Biol. 69, 1–12 10.1007/BF01871236 6811749

[B39] Kim J. Guan K. L. (2011). Amino acid signaling in TOR activation. Annu. Rev. Biochem. 80, 1001–1032 10.1146/annurev-biochem-062209-094414 21548787

[B40] Kurtzhals P. Schläffer L. Sorensen A. Kristensen C. Jonassen I. Schmid C. (2000). Correlations of receptor binding and metabolic and mitogenic potencies of insulin analogs desgined for clinical use. Diabetes 49, 1005–2000 10.2337/diabetes.49.6.999 10866053

[B41] Leslie N. R. Downes C. P. (2004). PTEN function: how normal cells control it and tumour cells lose it. Biochem. J. 382, 1–11 10.1042/BJ20040825 15193142 PMC1133909

[B42] Manning B. D. Cantley L. C. (2003). Rheb fills a GAP between TSC and TOR. Trends Biochem. Sci. 28, 573–576 10.1016/j.tibs.2003.09.003 14607085

[B43] Martin D. E. Hall M. N. (2005). The expanding TOR network. Curr. Opin. Cell Biol. 17, 158–166 10.1016/j.ceb.2005.02.008 15780592

[B44] Martin S. Zhang Z. Martino A. Faulon J.-L. (2007). Boolean dynamics of genetic regulatory networks inferred from microarray time series data. Bioinformatics 23, 866–874 10.1093/bioinformatics/btm021 17267426

[B45] McDowell H. E. Eyers P. A. Hundal H. S. (1998). Regulation of System A amino acid transport in L6 rat skeletal muscle cells by insulin, chemical and hyperthermic stress. FEBS Lett. 441, 15–19 10.1016/S0014-5793(98)01508-7 9877156

[B46] Montagne J. Stewart M. J. Stocker H. Kozma E. Kozma S. C. Thomas G. (1999). Drosophila S6 Kinase: a regulator of cell size. Science 285, 2126–2129 10.1126/science.285.5436.2126 10497130

[B47] Nassif N. T. Lobo G. P. Wu X. Henderson C. J. A. Morrison C. D. Eng C. (2004). PTEN mutations are common in sporadic microsatellite stable colorectal cancer. Oncogene 23, 617–628 10.1038/sj.onc.1207059 14724591

[B48] Nijhout H. F. Berg A. M. Gibson W. T. (2003). A mechanistic study of evolvability using the mitogen-activated protein kinase cascade. Evol. Dev. 5, 281–294 10.1046/j.1525-142X.2003.03035.x 12752767

[B49] Ogg S. Paradis S. Gottlieb S. Patterson G. I. Lee L. Tissenbaum H. A. (1997). The Fork head transcription factor DAF-16 transduces insulin-like metabolic and longevity signals in *C. elegans*. Nature 389, 994–999 10.1038/40194 9353126

[B50] Oldham S. Hafen E. (2003). Insulin/IGF and target of rapamycin signaling: a TOR de force in growth control. Trends Cell Biol. 13, 79–85 10.1016/S0962-8924(02)00042-9 12559758

[B51] Pal A. Barber T. M. Van de Bunt M. Rudge S. A. Zhang Q. Lachlan K. L. (2013). PTEN mutations as a cause of constitutie insulin sensitivity and obesity in human beings. Lancet 381, S13 10.1016/S0140-6736(13)60453-5 22970944 PMC4072504

[B53a] Puig O. Tijan R. (2005). Transcriptional feedback control of insulin recepetor by dFOXO/FOXO1. Genes Dev. 19, 2435–2446 10.1101/gad.1340505 16230533 PMC1257398

[B52] Puig O. Marr M. T. Ruhf M. L. Tijan R. (2003). Control of cell number of *Drosophila* FOXO: downstream and feedback regulation of the insulin receptor pathway. Genes Dev. 17, 2006–2020 10.1101/gad.1098703 12893776 PMC196255

[B53] Reinitz J. Sharp D. H. (1995). Mechanism of eve stripe formation. Mech. Dev. 49, 133–158 10.1016/0925-4773(94)00310-J 7748785

[B54] Sarbassov D. D. Ali S. M. Sabatini D. M. (2005). Growing roles for the mTOR pathway. Curr. Opin. Cell Biol. 15, 596–603 10.1016/j.ceb.2005.09.009 16226444

[B55] Sedaghat A. R. Sherman A. Quon M. J. (2002). A mathematical model of metabolic insulin signaling pathways. Am. J. Physiol. Endocrinol. Metab. 283, E1084–E1101 12376338 10.1152/ajpendo.00571.2001

[B56] Seger R. Krebs E. G. (1995). The MAPK signaling cascade. FASEB J. 9, 726–735 7601337

[B57] Shingleton A. W. Frankino W. A. (2013). New perspectives on the evolution of exaggerated traits. Bioessays 35, 100–107 10.1002/bies.201200139 23255216

[B58] Snell-Rood E. C. Moczek A. P. (2012). Insulin signaling as a mechanism underlying developmental plasticity: the role of FOXO in a nutritional polyphenism. PLoS ONE 7:e34857 10.1371/journal.pone.0034857 22514679 PMC3325941

[B59] So S. Miyahara K. Ohshima Y. (2011). Control of body size in *C. elegans* dependent on food and insulin/IGF-1 signal. Genes Cells 16, 639–651 10.1111/j.1365-2443.2011.01514.x 21501345

[B60] Song M. S. Salmena L. Pandolfi P. P. (2012). The functions and regulation of the PTEN tumour suppressor. Nat. Rev. Mol. Cell Biol. 13, 283–296 22473468 10.1038/nrm3330

[B61] Southgate R. J. Neill B. Prelovsek O. El-Osta A. Kamei Y. Miura S. (2007). FOXO1 regulates the expression of 4E-BP and inhibits mTOR signaling in mammalina skeletal muscle. J. Biol. Chem. 282, 21176–21186 10.1074/jbc.M702039200 17510058

[B62] Stagsted J. Hansen T. Roth R. A. Goldstein A. Olsson L. (1993). Correlation between insulin receptor occupancy and tyrosine kinase activity at low insulin concentrations and effect of major histocompatibility complex class I-derived peptide. J. Pharmacol. Exp. Ther. 267, 997–1001 8246175

[B63] Standaert M. L. Bandyopadhyay G. Kanoh Y. Sajan M. P. Farese R. V. (2001). Insulin and PIP3 activate PKC by mechanisms that are both dependent and independent of phosphorylation of activation loop (T410) and autophosphorylation (T560) sites. Biochemistry 40, 249–255 10.1021/bi0018234 11141077

[B64] Sutter N. B. Bustamante C. D. Chase K. Gray M. M. Zhao K. Zhu L. (2007). A single IGF1 allele is a major determinant of small size in dogs. Science 316, 112–115 10.1126/science.1137045 17412960 PMC2789551

[B65] Tang H. Y. Smith M. S. B. Driscoll M. V. Salhadar S. Shingleton A. W. (2011). FOXO regulates organ-specific phenotypic plasticity in *Drosophila*. PLoS Genetics 7:e1002373 10.1371/journal.pgen.1002373 22102829 PMC3213149

[B66] Taniguchi C. M. Emanuelli B. Kahn C. R. (2006). Critical nodes in signaling pathways: insights into insulin action. Nat. Rev. Mol. Cell Biol. 7, 85–96 10.1038/nrm1837 16493415

[B67] Tatar M. Bartke A. Antebi A. (2003). The endocrine regulation of aging by insulin-like signals. Science 299, 1346–1351 10.1126/science.1081447 12610294

[B68] Valovka T. Verdier F. Cramer R. Zhyvoloup A. Fenton T. Rebholz H. (2003). Protein kinase C phosphorylates ribosomal protein S6 kinase betaII and regulates its subcellular localization. Mol. Cell. Biol. 23, 852–863 10.1128/MCB.23.3.852-863.2003 12529391 PMC140705

[B69] Vinod P. K. U. Venkatesh K. V. (2009). Quantification of the effect of amino acids on an integrated mTOR and insulin signaling pathway. Mol. Biosyst. 5, 1163–1173 10.1039/b816965a 19756306

[B70] Vohradsky J. (2001). Neural network model of gene expression. FASEB J. 15, 846–854 10.1096/fj.00-0361com 11259403

[B71] Wolkenhauer O. Ullah M. Kolch W. Cho K.-H. (2004). Modeling and simulation of intracellular dynamics: choosing an appropriate framework. IEEE Trans. Nanobioscience 3, 200–207 10.1109/TNB.2004.833694 15473072

[B72] Zhao H. Cui Y. Dupont J. Sun H. Hennighausen L. Yakar S. (2005). Overexpression of the tumor suppressor gene phosphatase and tensin homologue partially inhibits wnt-1-induced mammary tumorigenesis. Cancer Res. 65, 6864–6873 10.1158/0008-5472.CAN-05-0181 16061670

